# The Ecological Impacts of Dry and Hot Shocks in the Land of Midnight Sun

**DOI:** 10.1111/gcb.70391

**Published:** 2025-08-01

**Authors:** Lianhong Gu, Bo Gao

**Affiliations:** ^1^ Environmental Sciences Division Oak Ridge National Laboratory Oak Ridge Tennessee USA

**Keywords:** Arctic, Arctic plant physiology, carbon storage and fluxes, drought, heatwave, permafrost

Contreras‐Serrano et al. (2025) and Heinzelmann et al. (2025) investigated how droughts and heatwaves affect plant physiology and community functions in the Arctic. At first glance, such studies may seem counterintuitive. As the Earth's refrigerator, the Arctic is probably the least likely place that one might associate with droughts and heatwaves. However, dry and hot shocks do occur there, and their frequency and intensity can be surprisingly high. On June 20, 2020, the thermometer at the weather station of the Siberian town of Verkhoyansk (67.55^o^ N and 133.40° E) witnessed a record temperature of 38°C in the Article Circle (Ciavarella et al. 2021). This record Arctic temperature was the consequence of the congruence of the powers of the stratospheric polar vortex, the tropospheric jet stream, and the Arctic Oscillation, all of which reached record strength in the early months of 2020, priming the conditions for an unprecedented ecological disturbance (Overland and Wang 2021). This extreme event, now known as the 2020 Siberian heatwave, caused widespread wildfires, insect outbreaks, and permafrost degradation, releasing almost 60 million tons of carbon into the atmosphere (ESOTC 2020). Extreme weather events like this are not just limited to Siberia (Dobricic et al. 2020). The Arctic has warmed up much faster than the rest of the world, that is, “Arctic Amplification” (Moon et al. 2024). The total land area affected by severe, extreme, and very extreme heatwaves has doubled, tripled, and quadrupled, respectively, across the terrestrial Arctic since the 1950s, and the extent is projected to continue increasing throughout the 21st century (Rantanen et al. 2024).

Why do we care? In a variety of ways, what happens in the Arctic doesn't stay in the Arctic. Droughts and heatwaves not only threaten local ecosystems but may also disrupt the Arctic's role as a planetary heat sink. In the lower latitudes of the Earth, particularly in the Tropics, the surface receives far more solar energy than it loses thermal energy to the atmosphere and space, whereas, in the higher latitude arctic regions, the reverse is true. This contrast allows the Arctic and the Tropics to act as the yin–yang partners of the Earth system. These two partners perform a delicate dance together to balance the Earth's surface energy budget: The Tropics send warm air and water polarward to chill, while the Arctic reciprocates by pushing cold air and water equatorward to warm up. This exchange forms the foundation of the global weather patterns, which we are familiar with in the midlatitudes. However, arctic droughts and heatwaves may disrupt this planetary circulation, leading to unexpected weather regimes in populated regions in the lower latitudes. Additionally, due to the historical accumulation and slow decomposition of organic matter in the Arctic, perennially frozen soil (permafrost) contains about twice as much carbon as in the atmosphere (Schuur et al. 2022). Increased occurrence of droughts and heatwaves may expose this massive amount of previously protected organic matter to microbial attacks, accelerating the release of carbon dioxide and methane into the atmosphere and further exacerbating the already severe impact of anthropogenic emissions. According to the 2024 NOAA report card, the arctic tundra is undergoing a shift from being a global carbon sink to a carbon source (Moon et al. 2024). Moreover, the positive feedback between arctic heatwaves and sea ice losses is of global consequence (Dobricic et al. 2020). Arctic heatwaves reduce the ocean area covered by ice, whereas the reduction in the iced ocean area decreases surface albedo and amplifies warming. This mutually reinforcing interaction can hasten arctic ice sheet melting and lead to a global rise in sea level.

As in the lower latitudes, the impacts of drought and heatwave start with plants, with subsequent effects cascading down the food chain and spreading from ecological to hydrological and atmospheric systems. However, for many reasons, the ecological impacts of drought and heatwave are expected to differ between the Arctic and lower latitudes. In the lower latitudes, with longer growing seasons, plants whacked by extreme events may get a second chance to develop, grow, and reproduce in the same season. In contrast, arctic plants have one chance to eke out a living in a very short growing season, and their life cycle in a whole year may be terminated by a single extreme event. Moreover, the dark nights of lower latitudes can provide plants with much‐needed stress relief during droughts and heatwaves. The midnight sun, however, may limit such relief for arctic plants. During daytime photosynthetic activities, plants produce reactive oxygen species (ROS). At low concentrations, ROS serve as signaling molecules that activate defense mechanisms against environmental stresses. However, at high concentrations, ROS can irreversibly damage cell membranes and organelles. To control this risk, plants use a complex scavenging system timed by a circadian clock to remove excess ROS so that the production and scavenging of ROS are in balance (ROS homeostasis, Jiménez et al. 2021). The midnight sun may make it harder for plants to achieve ROS homeostasis during extreme events. Thus, it would not be surprising if terrestrial arctic ecosystems in general, and plants in particular, are more sensitive to extreme events compared to those in the lower latitudes.

Contreras‐Serrano et al. (2025) and Heinzelmann et al. (2025), both conducted at the long‐term Abisko Scientific Research Station (68.35° N, 18.82° E), Sweden, contribute to the much‐needed understanding of the ecological impacts of drought and heatwave in the Arctic. Both studies used mesocosm approaches in their experiments. Contreras‐Serrano et al. (2025) focused on the impacts on photosynthesis and emissions of volatile organic compounds (VOC) of three widely distributed arctic shrub species ( *Betula nana* , *Empetrum hermaphroditum* , and *Salix* spp.) while Heinzelmann et al. (2025) were interested in the contrast in the drought responses of CO_2_ fluxes and shoot mortality between tundra heath and *Sphagnum* peatland plant communities. Contreras‐Serrano et al. (2025) found that heatwave decreased the photosynthesis of *B. nana* but not that of *E. hermaphroditum* and *Salix* spp. In contrast, drought reduced the photosynthesis of all three species. They also found that heatwave not only significantly increased the VOC emissions of all three species but also made the emission profile less diverse. This finding has implications for atmospheric chemistry and air quality during heatwaves. Heinzelmann et al. (2025) showed that for both tundra heath and *Sphagnum* peatland plant communities, drought suppressed gross primary productivity (GPP) and ecosystem respiration (ER) and the suppression on GPP was more than that on ER, reducing net carbon uptake in both ecosystems. In addition, they observed that drought led to substantial increases in dead shoots in both ecosystems. However, the impact of drought on *Sphagnum* peatland was less severe than on tundra heath in terms of both carbon fluxes and plant mortality. This difference was likely due to the pre‐drought water storage in spongy *Sphagnum* mosses. Taken together, the studies by Contreras‐Serrano et al. (2025) and Heinzelmann et al. (2025) suggest that, compared with ecosystems in warmer regions, arctic ecosystems compensate for lower species diversity with higher functional diversity, which is key to understanding the ecological impacts of drought and heatwave in the Arctic.

More in‐depth studies of drought and heatwave in the Arctic are needed. Mesocosm approaches such as those used in Contreras‐Serrano et al. (2025) and Heinzelmann et al. (2025) are convenient for controlling environmental conditions and for managing sample sizes. Abisko is within the discontinuous zone of permafrost, and neither of these two studies included permafrost dynamics in their experiments. However, permafrost is a key part of arctic ecosystems (Schuur et al. 2022) and is vulnerable to droughts and heatwaves (Dobricic et al. 2020). Permafrost thaw not only accelerates organic matter decomposition but also affects nutrient dynamics, geomorphology, and hydrology, with feedback on plant and microbial communities. To understand and predict accurately the ecological impacts of extreme events in the Arctic, we need in situ whole ecosystem manipulative experiments in which the active and inactive layers of the soil can interact and change depths, allowing an evaluation of how belowground dynamics may alleviate or augment the impacts of droughts and heatwaves. New experimental techniques that are more comprehensive and complex than mesocosm will need to be developed to overcome a wide range of engineering and eco‐hydrological challenges unique to permafrost regions.

## Author Contributions

**Lianhong Gu:** conceptualization, investigation, and Writing. **Bo Gao:** investigation and writing.

## Conflicts of Interest

The authors declare no conflicts of interest.

## Linked Articles

This article is a Commentary regarding Contreras‐Serrano et al, https://doi.org/10.1111/gcb.70187 and https://doi.org/10.1111/gcb.70210.

## Data Availability

Data sharing not applicable to this article as no datasets were generated or analysed for the current article.
